# The Pathological Pattern of Breast Cancer in the Najran Region of Saudi Arabia: A Retrospective Study

**DOI:** 10.7759/cureus.67621

**Published:** 2024-08-23

**Authors:** Abdulrahman M Alamri, Hajar H AlWadai, Saad M Almowallad, Saleh M AlKulayb, Zahra E Abdalwahab, Shuruq M Alzahuf, Saleh H Alshaiban

**Affiliations:** 1 Surgery, College of Medicine, Najran University, Najran, SAU; 2 Surgery, King Khalid Hospital, Najran, SAU; 3 Internal Medicine, Najran University, Najran, SAU; 4 Emergency Medicine, Najran University, Najran, SAU; 5 College of Medicine, Najran University, Najran, SAU

**Keywords:** saudi arabia, najran region, retrospective study, cancer, breast

## Abstract

Background

Breast cancer (BC) is the most widespread cancer on a global scale, and its prevalence is likewise significant in the Kingdom of Saudi Arabia. Nevertheless, the data accessible regarding the epidemiology and histopathological characteristics of BC in clinical practice is restricted and primarily confined to research endeavors.

Aim

This study aims to investigate the histopathological profile of women diagnosed with BC seeking treatment at King Khalid Hospital in the Najran region of the Kingdom of Saudi Arabia.

Methods

In this retrospective study, BC biopsies performed on Saudi patients at King Khalid Hospital between January 2018 and December 2022 were examined. All records of breast biopsies from this timeframe were extracted from the hospital’s histopathology laboratory computer database after written permission from the head of the laboratory department. For all neoplastic lesions, the World Health Organization’s 2012 categorization of breast tumors was applied.

Results

A total of 61 women with BC were included. Women’s age ranged from 30 to 89 years, with a mean age of 49.6 ± 12.3 years. The most reported BC was invasive ductal carcinoma (IDC; 70.5%). Other types reported included invasive papillary carcinoma (8.2%), ductal carcinoma in situ (4.9%), and invasive lobular carcinoma (3.3%). A total of 14 (23%) of the study women had multifocal cancer. Ki-67 was high in 19 cases (31.1%); six (9.8%) had BRCA1 mutations, and six (9.8%) had BRCA2 mutations.

Conclusion

The current study revealed that BC was frequent among young females, mainly IDC, which was reported on both sides at different sizes and grades. Breast lump was the most commonly presented symptom and had a high representation in women with hormonal receptors, mainly estrogen receptors, but positive genetic testing was infrequent.

## Introduction

Breast cancer (BC) is a pervasive and significant cause of mortality globally and is one of the most commonly diagnosed forms of cancer [1]. Reports indicate a higher incidence of BC-related deaths in transitioning regions like Western Africa, Polynesia, and the Caribbean, as opposed to more developed regions such as Australia, New Zealand, Western Europe, Northern America, and Northern Europe [2]. According to the World Health Organization (WHO), BC represents a substantial global health burden for women, estimated at 19.6 million disability-adjusted life years [3,4].

In Saudi Arabia, Alghamdi et al. documented 1152 cases of female BC in 2008, 1308 cases in 2009, and 1473 cases in 2010 [5]. According to WHO, the global statistics for BC in 2020 revealed 2.3 million women diagnosed and 685,000 deaths [3]. Projections suggest that by 2030, the annual global incidence of new cases may rise to 2.7 million, with the potential for nearly a million deaths [6]. In low- and medium-income countries, the surge in BC incidence is anticipated due to the adoption of Western lifestyles, including delayed pregnancies, reduced breastfeeding, early onset of menstruation, and inadequate dietary habits. This trend is further influenced by improved cancer registration and detection [7].

Various factors contribute to the susceptibility of women to BC, encompassing age, family history, and genetic mutations [8]. Disturbances in the physiological levels of endogenous sex hormones (estrogen or progesterone) elevate the risk of BC, affecting both premenopausal and postmenopausal women, with a particularly heightened risk for those undergoing menopause after the age of 50 [8-11]. Genetic factors, specifically mutations in the BC such as BRCA 1 and BRCA2 genes, account for 40% of hereditary BC cases [12]. Additionally, several other variables influence the development of BC, including the usage of hormonal contraceptives, postmenopausal hormone therapy, obesity, alcohol consumption, smoking, diabetes, and exposure to radiation [11].

It is imperative to adopt preventive measures for BC, emphasizing the development of effective protocols for early diagnosis and treatment. Essential components include general preventive behaviors and comprehensive screening programs aimed at minimizing the incidence of BC and facilitating early treatment [13]. Noteworthy progress in diagnostic tools and therapeutic interventions, guided by established protocols like those from the Breast Health Global Initiative (BHGI), has notably decreased BC mortality rates in developed nations and advanced BC control globally [13,14]. However, the available data on the epidemiology and histopathological characteristics of BC in clinical practice are limited and predominantly confined to research endeavors. Consequently, this study aims to investigate the profile of Saudi female patients diagnosed with BC who seek treatment at King Khalid Hospital in the Najran region of Saudi Arabia.

## Materials and methods

This retrospective study examined BC biopsies conducted on Saudi patients at King Khalid Hospital, spanning from January 2018 to December 2022. The study excluded non-Saudi patients and those with cutaneous basal cell carcinoma. Conducted primarily in the Histopathology Laboratory of King Khalid Hospital, the research involved retrieving all breast biopsy records reported within the specified timeframe from the laboratory’s computer database. Additionally documented were data relating to the tumor, such as laterality, multiplicity, lesion size, diagnosis, and concomitant diseases. Surgical specimens provided more information about multicentricity, skin/nipple involvement, the status of lymph nodes (LNs) (in the event of linked axillary procedures), and any history of recurrence that could be discovered. Individuals with multiple identical lesions, regardless of when they were excised, were counted only once, as were individuals with multiple specimens for the same lesion. For all neoplastic lesions, the World Health Organization’s 2012 categorization of breast tumors was applied (See Tables 1-3). Since the records were gathered from the histology laboratory, written approval was obtained from the head of the laboratory department.

**Table 1:** WHO breast tumor classifications

Histological type	Grade	Stage
Ductal carcinoma in situ (DCIS)	Not applicable (considered non-invasive)	Stage 0
Lobular carcinoma in situ (LCIS)	Not applicable (considered a marker for increased risk)	Stage 0
Invasive ductal carcinoma (IDC)	Grade 1 (well-differentiated) to grade 3 (poorly differentiated)	Stages I-IV
Invasive lobular carcinoma (ILC)	Grade 1 (well-differentiated) to grade 3 (poorly differentiated)	Stages I-IV
Mixed ductal-lobular carcinoma	Grade 1 (well-differentiated) to grade 3 (poorly differentiated)	Stages I-IV
Mucinous carcinoma	Varies	Varies
Tubular carcinoma	Varies	Varies
Medullary carcinoma	Varies	Varies
Papillary carcinoma	Varies	Varies
Other less common types	Varies	Varies

**Table 2:** Breast cancer grades

Grade	Description
I	Well-differentiated (low grade)
II	Moderately differentiated (intermediate grade)
III	Poorly differentiated (high grade)

**Table 3:** Stages of breast cancer

Stage	Description
0	Carcinoma in situ: abnormal cells are present but have not spread to nearby tissue
I	Early-stage breast cancer: where the tumor is small and localized to the breast
II	The tumor is larger or involves nearby lymph nodes but has not spread to distant parts of the body
III	Locally advanced cancer: where the tumor is larger and may have spread to nearby lymph nodes or tissues
IV	Metastatic cancer: where the cancer has spread to other parts of the body, such as the lungs, liver, bones, or brain; this is also known as advanced or stage 4 breast cancer

The data was collected, reviewed, and entered into IBM SPSS Statistics, version 21.0 (IBM Corp., Armonk, NY). An alpha threshold of 0.05 was used in a two-tailed test, with results considered significant if the p-value was less than or equal to 0.05. The descriptive analysis involved creating frequency distributions and percentages for various study variables, encompassing women’s bio-demographic details, cancer symptoms, types, pathological features, and the pathological stage of cancer. Crosstabulation for graphing distribution of the BC type by women’s age, breast size, and cancer side was done using chi-square and an exact probability test for small frequency distributions.

## Results

A total of 61 women with BC were included. The women ranged from 30 to 89 years, with a mean age of 49.6 ± 12.3 years. Only six women (9.8%) had a family history of BC. Regarding the cancer side, it was on the left side among 40 (65.6%) women, on the right side among 20 (32.8%), and bilateral in only one woman. Symptoms were experienced for less than six months among 38 (62.3%) women, six to 12 months among 16 (26.2%), and more than 12 months among seven (11.5%) women. The most reported symptoms included breast lump among 47 (81%) women, pain among 13 (22.4%) women, and nipple discharge among nine (15.5%) women (Table 4).

**Table 4:** Breast cancer types and pathological features among women attending King Khalid Hospital, Najran, Saudi Arabia The data has been represented as N (number of cases) and % (percentage of cases).

Cancer type and pathological features		No	%
Cancer type	Invasive ductal carcinoma	43	70.5%
	Invasive papillary carcinoma	5	8.2%
	Ductal carcinoma in situ	3	4.9%
	Invasive lobular carcinoma	2	3.3%
	Medullary carcinoma	2	3.3%
	Inflammatory cancer	1	1.6%
	Inflammatory fibroblastic tumor	1	1.6%
	Intraductal papilloma	1	1.6%
	Intraductal papilloma with foci	1	1.6%
	Mucinous cancer	1	1.6%
	Mucinous carcinoma	1	1.6%
Grade of cancer	Grade I	18	29.5%
	Grade II	25	41.0%
	Grade III	18	29.5%
Cancer size	<2 cm	21	34.4%
	2-5 cm	17	27.9%
	>5 cm	23	37.7%
Axillary lymph node	Yes	25	41.0%
	No	36	59.0%
Calcification	Yes	18	29.5%
	No	43	70.5%
Cancer margin	Positive	5	8.2%
	Negative	56	91.8%
Number of lymph node involvement	None	34	55.7%
	2-3 (N1)	13	21.3%
	4-9 (N2)	9	14.8%
	>10 (N3)	5	8.2%
Lymphovascular invasion	Yes	7	11.5%
	No	54	88.5%
Hormonal receptor; [estrogen]	Yes	40	65.6%
	No	21	34.4%
Hormonal receptor; [progesterone]	Yes	33	54.1%
	No	28	45.9%
Hormonal receptor; [HER2]	Yes	21	34.4%
	No	40	65.6%
Tumor necrosis	Yes	9	14.8%
	No	52	85.2%
Tumor extension	Yes	5	8.2%
	No	56	91.8%
Total number of samples		61	

The most reported BC was invasive ductal carcinoma (IDC) among 43 (70.5%) women, while other types were reported, including invasive papillary carcinoma among five (8.2%) women, which were encapsulated papillary tumors with invasion. They were low grade, with positive estrogen and progesterone hormonal receptors, and no LN metastasis. Others were ductal carcinoma in situ among three (4.9%) women, invasive lobular carcinoma among two (3.3%) women, and medullary carcinoma among two (3.3%) women. Twenty-five (41%) women had grade II cancer, which was less than 2 cm among 21 (34.4%) and more than 5 cm among 23 (37.7%). A total of 25 (41%) cases had axillary LNs, 18 (29.5%) had calcification, and the cancer margin was positive among five (8.2%). Thirty-four (55.7%) cases had no LN involvement, while 13 (21.3%) cases had the N1 stage (two to three LNs) LN involvement. Lymphovascular invasion was reported among seven (11.5%), 40 (65.6%) had estrogen receptors, 33 (54.1%) had progesterone receptors, and 21 (34.4%) had HER2. Exactly nine (14.8%) had tumor necrosis, and tumor extension was found among only five (8.2%) cases (Table 5).

**Table 5:** General health data of study women with breast cancer in King Khalid Hospital, Najran, Saudi Arabia The data has been represented as N (number of cases) and % (percentage of cases). The total number of patients was 61.

Bio-demographic data	No	%
Age in years		
<50	36	59.00%
>50	25	41.00%
Mean ± SD	49.6 ± 12.3	
Family history of breast cancer		
Yes	6	9.80%
No	55	90.20%
Breast side		
Right	20	32.80%
Left	40	65.60%
Bilateral	1	1.60%
Symptoms duration (months)		
<6 months	38	62.30%
6-12 months	16	26.20%
>12 months	7	11.50%
Symptoms		
Breast lump	47	81.00%
Breast pain	13	22.40%
Nipple discharge	9	15.50%
Nipple retraction	3	5.20%
Others	7	12.10%

A total of 14 (23%) of the study women had multifocal cancer. Ki-67, a protein pivotal in cell proliferation, identified through staining of tumor tissue samples, highlighting actively dividing cell nuclei, and crucial in BC diagnosis, was high among 19 (31.1%) of the study women. Ki-67 is interpreted as low (under 20%) and high (above 20-30%) by the percentage of cells positively stained. Six (9.8%) had BRCA1, and six (9.8%) had BRCA2. Mammogram suggestive cancer results were reported among 52 (85.2%), and ultrasound (US) suggestive cancer results were reported among 52 (85.2%). Twenty-three women (37.7%) had mammogram B1RADs V, and 18 (29.5%) had the same US B1RADs. Sentinel LN biopsy was positive among three (4.9%) women (Table 6).

**Table 6:** Pathological stage of breast cancer types and theological features among women attending King Khalid Hospital, Najran, Saudi Arabia The data has been represented as N (number of cases) and % (percentage of cases).

Pathological stage	No	%
Tumor focality		
Unifocal	47	77.00%
Multifocal	14	23.00%
Ki-67		
None	37	60.70%
Low	5	8.20%
High	19	31.10%
Genetic testing; [BRCA1]		
Positive	6	9.80%
Negative	9	14.80%
Not done	46	75.40%
Genetic testing; [BRCA2]		
Negative	6	9.80%
Not done	55	90.20%
Mammogram report; [suggestive of cancer]		
Yes	52	85.20%
No	9	14.80%
Ultrasound report; [suggestive of cancer]		
Yes	52	85.20%
No	9	14.80%
Mammogram B1RADs		
Not reported	19	31.10%
I	1	1.60%
IV	9	14.80%
V	23	37.70%
VI	9	14.80%
Ultrasound B1RADs		
Not reported	27	44.30%
I	1	1.60%
IV	12	19.70%
V	18	29.50%
VI	3	4.90%
Sentinel lymph node biopsy		
Positive	3	4.90%
Negative	3	4.90%
Not done	55	90.20%

In continuation, findings also revealed that IDC was most reported among all women, both those below the age of 50 years (24, 66.7%) and above the age of 50 years (17, 68%), while invasive papillary carcinoma was more common among women aged more than 50 years (3, 12%) compared to women below the age of 50 years (2, 5.6%). Ductal carcinoma in situ and invasive lobular carcinoma were more frequent among women aged less than 50 years (2, 5.6% for each). All IDC (43, 100%) was on the right side, and so was half of the invasive lobular carcinoma (1, 50%), two (40%) of invasive papillary carcinomas, and one (33.3%) of ductal carcinoma in situ. All other types were on the left side. All intraductal papillomas with foci and inflammatory cancer were less than 2 cm. Also, all medullary carcinomas, intraductal papillomas, and mucinous carcinomas were more than 5 cm. IDCs were presented in different sizes; 19 (44.2%) were <2cm, 10 (23.2%) were from 2 to 5 cm, and 14 (32.6%) were >5 cm.

## Discussion

Analyzing patterns of diseases equips policymakers and health administrators to anticipate emerging and potential challenges [15]. Globally, BC stands as the predominant cancer impacting women, affecting one in 10 women across the world during their lifetime [16]. Within Saudi Arabia, BC ranks as the most prevalent malignancy, impacting approximately 127 of every 100,000 Saudi females. It constitutes 21.8% of all malignancies specific to age and gender among Saudi women [17].

The purpose of the current study was to ascertain the histopathological profile of BC patients who were receiving care at King Khalid Hospital. The study revealed that most of the affected women were aged less than 50 years, and most of them had unilateral BC presented mainly by breast lumps with breast pain. As for cancer type, IDC was the dominant type present in 43 (70.5%) women, while other types, such as invasive papillary carcinoma, ductal carcinoma in situ, and invasive lobular carcinoma, were also reported. IDC was presented nearly equally among women below and others above the age of 50 years and also on the right and left sides. However, intraductal papilloma was mainly present in women below the age of 50 years. Similar findings were reported in the literature, as BC has been found to affect women of different ages in different regions. For instance, in Lagos, Nigeria, the highest incidence of BC was found in women aged 31-40 years [18]. In South Yemen, women aged 40-49 years were more prone to developing BC [19]. Research conducted in Saudi Arabia indicated that the average age of onset for BC was 48.6 [20]. A thorough literature review encompassing Arab countries demonstrated that BC was diagnosed approximately a decade earlier, with the mean age being 48 years and one-third of patients falling below the age of 50 [21]. Similarly, an epidemiological study in Saudi Arabia concluded that BC is diagnosed in a notably younger age group, with 38.6% in the 30-44 age range and 31.2% in the 45-59 age range [22]. These collective findings signify that BC is identified at a younger age in the Arab region than the Western population.

According to the findings of the present study, invasive papillary carcinoma was mainly diagnosed above the age of 50 years and more on the left side. The most invasive ductal and lobular carcinomas were sized up to 5 cm, but invasive papillary carcinomas were sized 2-5 cm or more. Most cancers were stage II; less than half of the cases had axillary LNs, and less than one-third had calcifications. Lymphovascular invasion was detected among very few cases (nearly 12%). The findings also revealed that about two-thirds of the cases showed positive results for estrogen receptors and about half for progesterone receptors, but about one-third showed positive results for HER2. About three-fourths of the cases had unifocal tumors, with positive genetic testing among about 10%. Similar findings were reported by Amin et al. [23], wherein infiltrating ductal carcinoma was identified as the main lesion in 62.1% of cases diagnosed prior to the age of 50.

In Saudi Arabia, IDC was shown to be the most common kind of BC [24], making up 85.2% of cases in another study [25], collaborating with the findings of this present study. Albasri and colleagues showed that 2.7% of cases had invasive lobular carcinoma, and 8% of cases had ductal carcinoma in situ [25]. They also revealed that Saudi women with IDC were 46.9 years old on average, and of the patients who received an IDC diagnosis, 61.1% had tumors bigger than 2.5 cm, and 48.7% had grade III tumors. In continuation, of the patients, 57.1% had axillary nodal metastases, and 64.1% who had axillary nodal dissection had lymphovascular invasion [25]. Another study carried out in Pakistan reported differing rates (11.8%) of IDC among cases of BC [26]. In Central KSA, Al-Rikabi and Husain reported the highest percentage of IDC at 96.1%, with a smaller group comprising other types of malignant carcinomas [20]. In the Western region, Jamal’s study from Jeddah noted 88% IDC and 4.5% lobular carcinoma [27]. Inflammatory lesions comprised 11% of all lesions, with a mean age of 35.0 years [27]. In addition, Razik et al. discovered that, similar to the results of the current study, the most often reported symptom was a breast lump, followed by pain [28]. Their study also showed that ductal/lobular/papillary carcinoma presentations were less common in people between the ages of 31 and 45 than in people under 30, suggesting that younger women were more likely to be affected. However, in their study, Razik and colleagues revealed that fibroadenoma was diagnosed histopathologically in more than half of their patients [28].

Limitations of the study

While this study offers valuable insights into the histopathological patterns of BC in the Najran region of Saudi Arabia, it is important to acknowledge certain limitations. Firstly, the study relied on data from hospital records, which could have introduced biases due to potential inaccuracies or missing information, particularly for cases that were excluded. This reliance on secondary data limits the control over data quality and completeness. Secondly, the relatively small sample size of 61 cases restricts the statistical power of the findings, potentially limiting their generalizability to the broader population in Saudi Arabia or other regions. The limited sample size also reduces the ability to detect less common histopathological subtypes or variations in tumor characteristics.

Furthermore, the study was conducted at a single institution, King Khalid Hospital, which may not fully capture the diversity of BC presentations across the entire Najran region or other parts of the country. This single-center approach may lead to overrepresenting specific patient demographics or healthcare practices unique to this facility, potentially skewing the results. Future research with larger, multi-center studies would be beneficial to confirm these findings and provide a more comprehensive understanding of BC in this region.

## Conclusions

In conclusion, the current study revealed that BC was frequent among young females, mainly IDC, which was reported on both sides at different sizes and grades. Breast lump was the most presented symptom and high representation for women with hormonal receptors, mainly estrogen receptors, but positive genetic testing was infrequent. Most cases showed high BIRADS for both US and mammograms. It is highly recommended that a large-scale study, including all regions of the Kingdom of Saudi Arabia, be conducted to identify specific etiological factors and compare them to other Arab and Western countries.
